# Supplementary material for: Identifying genetic variants associated with ritodrine-induced pulmonary edema
Source: PLoS One. 2020 Nov 9;15(11):e0241215. doi: 10.1371/journal.pone.0241215 (PMC7652239; doi:10.1371/journal.pone.0241215)
Supplement: S1 Fig — (DOCX) [file pone.0241215.s001.docx]

**S1 Fig. Schematic of the data analysis steps.**

**
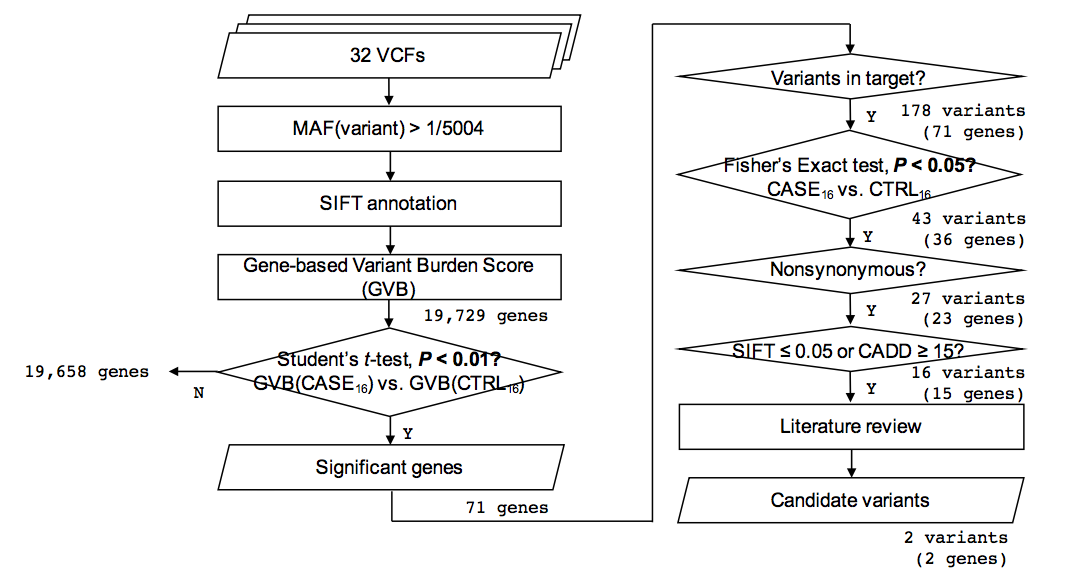
**

VCF, Variant Call Format; MAF, Minor Allele Frequency; SIFT, Sorting Intolerant From Tolerant; CADD, Combined Annotation Dependent Depletion.
