# Supplementary material for: Identifying genetic variants associated with ritodrine-induced pulmonary edema
Source: PLoS One. 2020 Nov 9;15(11):e0241215. doi: 10.1371/journal.pone.0241215 (PMC7652239; doi:10.1371/journal.pone.0241215)

**S5 Fig. Results of Sanger sequencing of *ADRA1A* (rs2229126) for the individuals without ritodrine-induced pulmonary edema.**


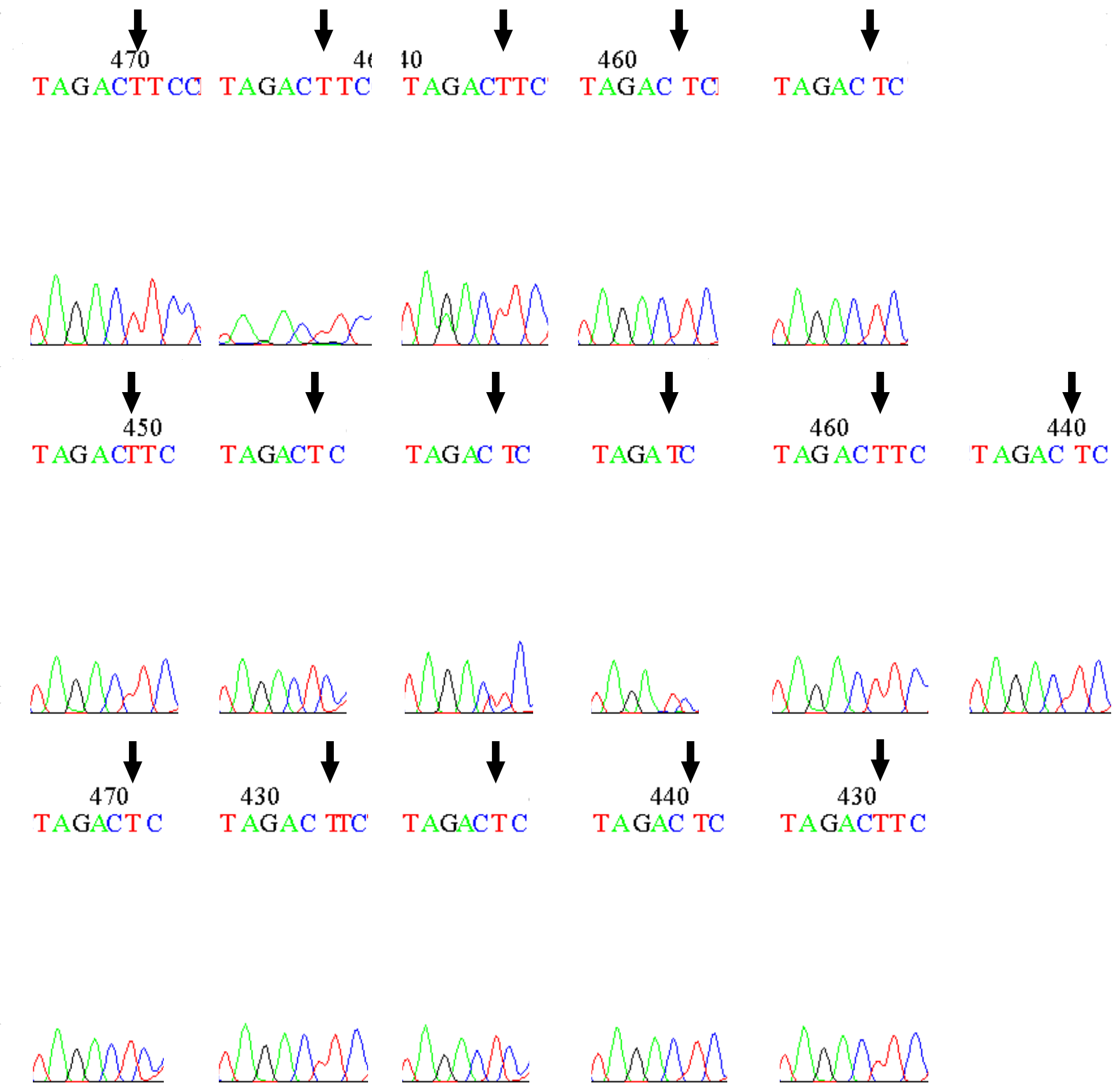

Supplement: S5 Fig — (DOCX) [file pone.0241215.s005.docx]
