# Supplementary material for: Identifying genetic variants associated with ritodrine-induced pulmonary edema
Source: PLoS One. 2020 Nov 9;15(11):e0241215. doi: 10.1371/journal.pone.0241215 (PMC7652239; doi:10.1371/journal.pone.0241215)
Supplement: S1 Table — (DOCX) [file pone.0241215.s006.docx]

**S1 Table. Frequency distribution for 16 deleterious variants significantly associated with ritodrine induced pulmonary edema.**

| Gene (rsID) | SIFT | CADD | Group | Allelic Frequency | | | | REF | HET | HOM | Dominant model | | Recessive model | | CATT |
| --- | --- | --- | --- | --- | --- | --- | --- | --- | --- | --- | --- | --- | --- | --- | --- |
|  |  |  |  | REF | ALT | P | OR (95% CI) |  |  |  | P | OR (95% CI) | P | OR (95% CI) | P |
| CPT2 (rs2229291) | 0.00 | 24.40 | Pulmonary Edema (+), N=16 | 17 | 15 | 0.014 | 4.6 (1.3-19.0) | 5 | 7 | 4 | 0.076 | 4.6 (0.9-28) | 0.101 | Inf (0.7-Inf) | 0.018 |
|  |  |  | Matched Control, N=16 | 27 | 5 |  |  | 11 | 5 | 0 |  |  |  |  |  |
| ADRA1A (rs2229126) | 0.59 | 18.55 | Pulmonary Edema (+), N=16 | 27 | 5 | 0.053 | Inf (1.0-Inf) | 11 | 5 | 0 | 0.043 | Inf (1.1-Inf) | 1 | 0 (0-Inf) | 0.080 |
|  |  |  | Matched Control, N=16 | 32 | 0 |  |  | 16 | 0 | 0 |  |  |  |  |  |
| PCSK9 (rs11583680) | 0.38 | 18.29 | Pulmonary Edema (+), N=16 | 31 | 1 | 0.053 | 0.12 (0-1) | 15 | 1 | 0 | 0.037 | 0.09  (0-0.8) | 1 | 0 (0-Inf) | 0.052 |
|  |  |  | Matched Control, N=16 | 25 | 7 |  |  | 9 | 7 | 0 |  |  |  |  |  |
| NUP210 (rs2280084) | 0.40 | 16.72 | Pulmonary Edema (+), N=16 | 20 | 12 | 0.045 | 0.32 (0.1-1.0) | 8 | 4 | 4 | 0.054 | 0.15  (0-1) | 0.458 | 0.44 (0.1-2.4) | 0.049 |
|  |  |  | Matched Control, N=16 | 11 | 21 |  |  | 2 | 7 | 7 |  |  |  |  |  |
| H1FOO (rs118098064) | 0.05 | 1.46 | Pulmonary Edema (+), N=16 | 25 | 7 | 0.148 | 4.1 (0.7-44) | 9 | 7 | 0 | 0.037 | 10.8 (1.1-558.0) | 1 | 0 (0-39) | 0.147 |
|  |  |  | Matched Control, N=16 | 30 | 2 |  |  | 15 | 0 | 1 |  |  |  |  |  |
| ASZ1 (rs1029396) | 0.13 | 22.50 | Pulmonary Edema (+), N=16 | 9 | 23 | 0.011 | 4.2 (1.3-14) | 1 | 7 | 8 | 0.037 | 11.7 (1.2-111.0) | 0.135 | 4.3 (0.9-21.3) | 0.011 |
|  |  |  | Matched Control, N=16 | 20 | 12 |  |  | 7 | 6 | 3 |  |  |  |  |  |
| LIPM (rs117982455) | 0.09 | 21.30 | Pulmonary Edema (+), N=16 | 26 | 6 | 0.024 | Inf (1.3-Inf) | 10 | 6 | 0 | 0.018 | Inf (1.5-Inf) | 1 | 0 (0-Inf) | 0.042 |
|  |  |  | Matched Control, N=16 | 32 | 0 |  |  | 16 | 0 | 0 |  |  |  |  |  |
| HPS5 (rs7128017) | 0.01 | 23.50 | Pulmonary Edema (+), N=16 | 23 | 9 | 0.013 | 11.7 (1.5-546.8) | 8 | 7 | 1 | 0.015 | 15 (1.6-142.2) | 1 | 0 (0-Inf) | 0.022 |
|  |  |  | Matched Control, N=16 | 31 | 1 |  |  | 15 | 1 | 0 |  |  |  |  |  |
| NAALADL1 (rs12223986) | 0.03 | 24.80 | Pulmonary Edema (+), N=16 | 20 | 12 | 0.005 | 8.7 (1.7-88) | 6 | 8 | 2 | 0.009 | 11 (1.6-129) | 0.484 | Inf (0.2-Inf) | 0.009 |
|  |  |  | Matched Control, N=16 | 30 | 2 |  |  | 14 | 2 | 0 |  |  |  |  |  |
| TBC1D21 (rs16958445) | 0.24 | 15.21 | Pulmonary Edema (+), N=16 | 26 | 6 | 0.024 | Inf (1.3-Inf) | 10 | 6 | 0 | 0.018 | Inf (1.5-Inf) | 1 | 0 (0-Inf) | 0.042 |
|  |  |  | Matched Control, N=16 | 32 | 0 |  |  | 16 | 0 | 0 |  |  |  |  |  |
| COMMD2 (rs9843784) | 0.31 | 17.35 | Pulmonary Edema (+), N=16 | 23 | 9 | 0.043 | 5.7 (1-59) | 7 | 9 | 0 | 0.023 | 8.3 (1.2-100) | 1 | 0 (0-Inf) | 0.030 |
|  |  |  | Matched Control, N=16 | 30 | 2 |  |  | 14 | 2 | 0 |  |  |  |  |  |
| COMMD2 (rs41267879) | 0.00 | 5.24 | Pulmonary Edema (+), N=16 | 23 | 9 | 0.043 | 5.7 (1-59) | 8 | 7 | 1 | 0.054 | 6.6 (1-78) | 1 | 0 (0-Inf) | 0.047 |
|  |  |  | Matched Control, N=16 | 30 | 2 |  |  | 14 | 2 | 0 |  |  |  |  |  |
| S100Z (rs1320308) | 0.08 | 23.1 | Pulmonary Edema (+), N=16 | 21 | 11 | 0.032 | 4.9 (1.1-31) | 8 | 5 | 3 | 0.054 | 6.6 (1-78) | 0.600 | 3.3 (0.2-193) | 0.045 |
|  |  |  | Matched Control, N=16 | 29 | 3 |  |  | 14 | 1 | 1 |  |  |  |  |  |
| RP11-257K9.8 (rs6927398) | 0.00 | 0.002 | Pulmonary Edema (+), N=16 | 19 | 13 | 0.022 | 4.7 (1.2-23) | 6 | 7 | 3 | 0.073 | 4.7 (0.9-31) | 0.226 | Inf (0.4-Inf) | 0.027 |
|  |  |  | Matched Control, N=16 | 28 | 4 |  |  | 12 | 4 | 0 |  |  |  |  |  |
| EPPK1 (rs13260439) | 0.07 | 20.3 | Pulmonary Edema (+), N=16 | 22 | 10 | 0.022 | 6.6 (1.2-68) | 7 | 8 | 1 | 0.023 | 8.3 (1.2-100) | 1 | Inf (0-Inf) | 0.024 |
|  |  |  | Matched Control, N=16 | 30 | 2 |  |  | 14 | 2 | 0 |  |  |  |  |  |
| RAD23B (rs1805329) | 0.28 | 23.3 | Pulmonary Edema (+), N=16 | 22 | 10 | 0.022 | 6.6 (1.2-68) | 8 | 6 | 2 | 0.054 | 6.6 (1-78) | 0.484 | Inf (0.2-Inf) | 0.036 |
|  |  |  | Matched Control, N=16 | 30 | 2 |  |  | 14 | 2 | 0 |  |  |  |  |  |
