# Supplementary material for: Identifying genetic variants associated with ritodrine-induced pulmonary edema
Source: PLoS One. 2020 Nov 9;15(11):e0241215. doi: 10.1371/journal.pone.0241215 (PMC7652239; doi:10.1371/journal.pone.0241215)
Supplement: S2 Table — (DOCX) [file pone.0241215.s007.docx]

**S2 Table. Variants significantly associated with ritodrine-induced pulmonary edema.**

| Gene | LR coefficients (β)  (95% CI) | LR *p* |
| --- | --- | --- |
| *CPT2 (rs2229291)* | 1.368 (0.1-2.9) | 0.048 |
| *ADRA1A (rs2229126)* | 18.615 (-273-Inf) | 0.995 |

Data were adjusted for cesarean delivery; CI, Confidence Interval, LR, Logistic Regression.
