# Supplementary material for: Identifying genetic variants associated with ritodrine-induced pulmonary edema
Source: PLoS One. 2020 Nov 9;15(11):e0241215. doi: 10.1371/journal.pone.0241215 (PMC7652239; doi:10.1371/journal.pone.0241215)
Supplement: S3 Table — (DOCX) [file pone.0241215.s008.docx]

**S3 Table. Distribution of coding variants in *ADRA1A* and *CPT2*.**

|  | **Gene** | *ADRA1A* | *ADRA1A* | *CPT2* | *CPT2* | *CPT2* |
| --- | --- | --- | --- | --- | --- | --- |
|  | **rsID** | rs2229126 | rs1048101 | rs2229291 | rs1799821 | rs1799822 |
|  | **Ref** | T | A | T | G | A |
|  | **Alt** | A | G | G | A | G |
| **Group** | **ID \ AA** | p.Glu465Asp | p.Cys347Arg | p.Phe352Cys | p.Val368Ile | p.Met647Val |
| PE case | Rit_001 | HET | HOM | . | HET | HET |
| PE case | Rit_002 | . | HOM | HOM | HOM | . |
| PE case | Rit_003 | . | HET | HET | HOM | HET |
| PE case | Rit_004 | . | HET | HET | HOM | . |
| PE case | Rit_005 | . | HOM | . | HET | . |
| PE case | Rit_006 | . | HOM | HOM | HOM | . |
| PE case | Rit_007 | . | HET | . | HET | HET |
| PE case | Rit_008 | HET | HOM | HET | HET | . |
| PE case | Rit_009 | . | HOM | HOM | HOM | . |
| PE case | Rit_010 | . | HOM | HET | HOM | . |
| PE case | Rit_011 | HET | HOM | . | HOM | . |
| PE case | Rit_012 | . | HOM | HET | HOM | HET |
| PE case | Rit_013 | HET | HOM | HET | HOM | . |
| PE case | Rit_014 | . | HOM | . | HET | . |
| PE case | Rit_015 | . | HOM | HOM | HOM | . |
| PE case | Rit_016 | HET | HOM | HET | HOM | . |
| Control | Rit_101 | . | HOM | . | HOM | . |
| Control | Rit_102 | . | HOM | . | HET | . |
| Control | Rit_103 | . | HET | HET | HOM | . |
| Control | Rit_104 | . | HOM | . | HET | . |
| Control | Rit_105 | . | HOM | . | HOM | HET |
| Control | Rit_106 | . | HET | . | HOM | . |
| Control | Rit_107 | . | HOM | . | HOM | . |
| Control | Rit_108 | . | HET | . | HOM | . |
| Control | Rit_109 | . | HOM | . | HOM | HET |
| Control | Rit_110 | . | HOM | . | HOM | . |
| Control | Rit_111 | . | HOM | . | HOM | . |
| Control | Rit_112 | . | HET | HET | HET | . |
| Control | Rit_113 | . | HET | . | HET | . |
| Control | Rit_114 | . | HOM | HET | HOM | . |
| Control | Rit_115 | . | HOM | HET | HET | . |
| Control | Rit_116 | . | HOM | . | HET | . |

PE, Pulmonary Edema; AA, Amino acid change; HET, Heterozygous; HOM, Homozygous.
